# A prominent air pollutant, Indeno[1,2,3-cd]pyrene, enhances allergic lung inflammation via aryl hydrocarbon receptor

**DOI:** 10.1038/s41598-018-23542-9

**Published:** 2018-03-26

**Authors:** Tzu-Hsuan Wong, Chon-Lin Lee, Hsiang-Han Su, Chin-Lai Lee, Chao-Chien Wu, Chin-Chou Wang, Chau-Chyun Sheu, Ruay-Sheng Lai, Sum-Yee Leung, Chi-Cheng Lin, Yu-Feng Wei, Chien-Jen Wang, Yu-Chun Lin, Hua-Ling Chen, Ming-Shyan Huang, Jeng-Hsien Yen, Shau-Ku Huang, Jau-Ling Suen

**Affiliations:** 1 Graduate Institute of Medicine, College of Medicine, Kaohsiung Medical University, Kaohsiung, Taiwan; 2 Department of Marine Environment and Engineering, National Sun Yat-sen University, Kaohsiung, Taiwan; 3 Research Center of Environmental Medicine, Kaohsiung Medical University, Kaohsiung, Taiwan; 4 Division of Pulmonary and Critical Care Medicine, Department of Internal Medicine, Kaohsiung Chang Gung Memorial Hospital and College of Medicine, Chang Gung University, Kaohsiung, Taiwan; 5 Department of Public Health, Kaohsiung Medical University, Kaohsiung, Taiwan; 6 Divison of Pulmonary and Critical Care Medicine, Department of Internal Medicine, Kaohsiung Medical University Hospital, Kaohsiung, Taiwan; 7 College of Medicine, Kaohsiung Medical University, Kaohsiung, Taiwan; 8 Division of Chest Medicine, Kaohsiung Veterans General Hospital, Kaohsiung, Taiwan; 9 Chest Division, Department of Internal Medicine, Antai Medical Care Cooperation Antai Tian-Sheng Memorial Hospital, Ping-Tung, Taiwan; 10 Division of Chest Medicine, Department of Internal Medicine, E-Da Hospital, I-Shou University, Kaohsiung, Taiwan; 11 National Institute of Environmental Health Sciences, National Health Research Institutes, Miaoli, Taiwan; 12 Division of Rheumatology, Department of Internal Medicine, Kaohsiung Medical University Hospital, Kaohsiung, Taiwan; 13 Lou-Hu Hospital, Shen-Zhen University, Shen-Zhen, China; 14 Department of Medicine, Johns Hopkins University School of Medicine, Baltimore, Maryland USA; 15 Department of Medical Research, Kaohsiung Medical University Hospital, Kaohsiung, Taiwan

## Abstract

Chronic exposure to ambient polycyclic aromatic hydrocarbons (PAHs) is associated with asthma, but its regulatory mechanisms remain incompletely defined. We report herein that elevated levels of urinary 1-hydroxypyrene, a biomarker of PAH exposure, were found in asthmatic subjects (*n* = 39) as compared to those in healthy subjects (*n* = 43) living in an industrial city of Taiwan, where indeno[1,2,3-cd]pyrene (IP) was found to be a prominent PAH associated with ambient PM_2.5_. In a mouse model, intranasal exposure of mice with varying doses of IP significantly enhanced antigen-induced allergic inflammation, including increased airway eosinophilia, Th2 cytokines, including IL-4 and IL-5, as well as antigen-specific IgE level, which was absent in dendritic cell (DC)-specific aryl hydrocarbon receptor (AhR)-null mice. Mechanistically, IP treatment significantly altered DC’s function, including increased level of pro-inflammatory IL-6 and decreased generation of anti-inflammatory IL-10. The IP’s effect was lost in DCs from mice carrying an AhR-mutant allele. Taken together, these results suggest that chronic exposure to environmental PAHs may pose a significant risk for asthma, in which IP, a prominent ambient PAH in Taiwan, was shown to enhance the severity of allergic lung inflammation in mice through, at least in part, its ability in modulating DC’s function in an AhR-dependent manner.

## Introduction

Asthma is a common chronic airway inflammatory disease characterized by airway obstruction, bronchial hyper-responsiveness and airway inflammation^1^. The increasing prevalence of asthma in last decades is an important public health issue in the developed countries^2^. Particularly in Taiwan, a recent survey of 24,999 first-grade elementary school students showed a significant high prevalence of physician-diagnosed atopic eczema, allergic rhinitis, and asthma, with 29.8%, 33.7% and 13%, respectively^3^. This alarming increase in prevalence highlights an urgent need for better understanding of the etiology and its causative mechanisms.

According to WHO estimates, more than 300 million people are suffered from asthma and air pollution has been considered as one important environmental risk for asthma^4,5^. Polycyclic aromatic hydrocarbons (PAHs), primarily associated with ambient particulate matters (PMs), are a group of chemicals that can induce oxidative stress and cytotoxicity^6^. They are usually generated by incomplete combustion of organic materials, such as coal, gas, and oil as well as garbage^7^. PAHs may enter human body through inhalation with fine PMs in air or ingestion with over-roasted foods^8^. Due to their lipophilic property, PAHs are easily absorbed by all routes of exposure and then metabolized into hydroxylated and glucuronide metabolites^9^. The most widely used urinary PAH metabolites as biomarkers for recent exposure are 1-hydroxypyrene (OH-Py) and 1-hydroxypyrene-O-glucuronide^10^. Exposure to PAHs has been associated with pulmonary diseases, such as allergic asthma, particularly in children^11^.

Aryl hydrocarbon receptor (AhR), a ligand-activated transcription factor from the Per-Arnt-Sim superfamily, acts as a cellular chemical sensor linking environmental pollutants and immunity. AhR is originally discovered as a high affinity receptor for 2,3,7,8-tetrachlorodibenzo-p-dioxin (TCDD); however, it also has been recognized as a receptor for many environmental pollutants, including benzo[a]pyrene (BaP). They can directly bind to AhR and trigger its translocation into nucleus to mediate the downstream effects, including detoxification and other cellular responses^12^. Some studies also reveal the role of AhR signaling in carcinogenesis^13,14^. Accumulated evidence suggests that while the experimental outcomes appear to vary depending, in part, on the dosage used, the AhR-ligand axis is now considered to play a role in the regulation of both innate and adaptive immune response. For example, our recent evidence suggests an important role of the AhR-ligand axis in controlling cellular homeostasis, maturation and optimal activation of several regulatory cell types, including macrophages and mast cells^15–17^. Notably, the ligand-AhR axis is shown to be critical in controlling the growth and function of mast cells in a calcium (Ca^2+^)- and reactive oxygen species (ROS)-dependent fashion^15,16^.

Dendritic cells (DCs) are the key antigen-presenting cell type for instructing T cell-mediated inflammation. Bone marrow-derived dendritic cells (BM-DCs) and T helper 17 (Th17) cells express high levels of AhR^18,19^. One previous study showed that BaP, a well-studied PAH, is able to inhibit the maturation, endocytic activity, IL-12 secretion, and T-cell stimulatory activity of human monocyte-derived DC *in vitro*^20^. Also, it has been shown that AhR promotes IL-22 production of Th17 cells^18^ and controls Foxp3 expression of regulatory T cells^21^. Further, AhR activation by prototypical ligands, TCDD and formylindolo[3,2-b]carbazole (FICZ), was shown to modulate DCs to display tolerogenic phenotypes which may facilitate Foxp3^+^ regulatory T cell differentiation^22^. AhR signaling also crosstalks with NF-κB and estrogen receptor signaling pathways, which are known to be important in regulating immune system^23^. A recent study shows AhR deficiency in DC leads to the perturbation of intestinal epithelium development and intestinal immunity^24^. However, the causal relationship between individual PAH and allergic asthma and the detailed mechanism still need to be elucidated.

Due to the alarming high prevalence of childhood allergic diseases in Taiwan^3^, we aimed to identify the main component of ambient PAHs and explored its pathogenic role in the development of allergic asthma. In this study, we demonstrated that indeno[1,2,3-cd]pyrene (IP) was the major component of ambient PAHs in Kaohsiung City in Taiwan, and that IP exposure exacerbated antigen-induced pulmonary inflammation in a mouse model of asthma and the IP’s effect was, at least in part, mediated by its impact on DC’s function in an AhR-dependent manner.

## Results

### IP was a prominent PAH associated with ambient PM_2.5_

**Figure 1:**
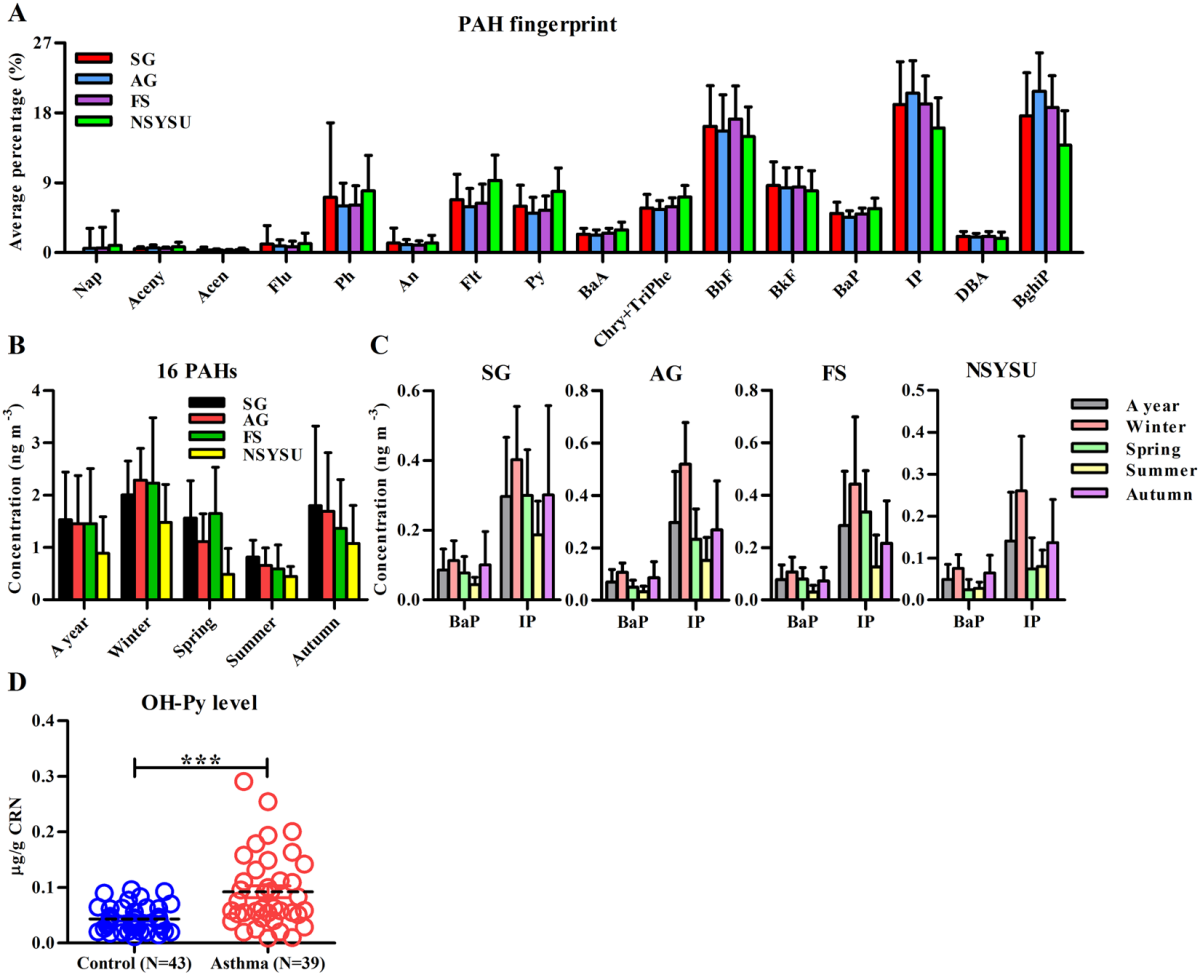
Analysis of ambient PAHs in southern Taiwan and of a surrogate uninary metabolite in a case-control study. (**A**) Normalized PAH composition distributions (mean ± SD) in PM_2.5_ in air from the four sampling sites. The description of the sampling sites and the full name of each PAH were summarized in Methods. (**B**) The seasonal variations of 16 PAHs (mean ± SD) in ambient PM_2.5_ in each site. (**C**) The difference of PM_2.5_ bound BaP and IP concentrations (mean ± SD) in each site and each season. (**D**) Analysis of urinary PAH metabolite, 1-hydroxypyrene (OH-Py), in asthma patients and controls. The levels of normalized urinary OH-Py by the levels of urinary creatinine (CRN) were measured in those with current asthma versus normal subjects, all of whom were non-smokers. Each circle represents each individual result. The line within the vertical points marks the mean for each group. ***P = 0.0001 by Mann-Whitney U test.

After monthly samplings of atmospheric PM_2.5_ in 4 monitoring stations in a subtropical industrial city Kaohsiung, 16 PAHs (USEPA priority) were analyzed. Results showed that among the 16 PAHs analyzed, IP was a prominent PAH associated with ambient PM_2.5_ in all sites (Fig. 1A) and all seasons (data not shown). The concentrations of PAHs were higher in winter and autumn than in summer (Fig. 1B). It is also noted that the average concentrations of IP was around four times higher than that of BaP in all sites and all seasons (Fig. 1C). In a case-control design, the urinary concentration of OH-Py, a major metabolite of pyrene and widely used as a marker of exposure to PAHs, was significantly higher in subjects with asthma than those in control subjects (mean ± SEM, 0.092 ± 0.011 μg/g creatinine (CRN) versus 0.043 ± 0.003 μg/g CRN, *P < *0.0001).

### IP exposure potentiated antigen-induced pulmonary allergic inflammation

**Figure 2:**
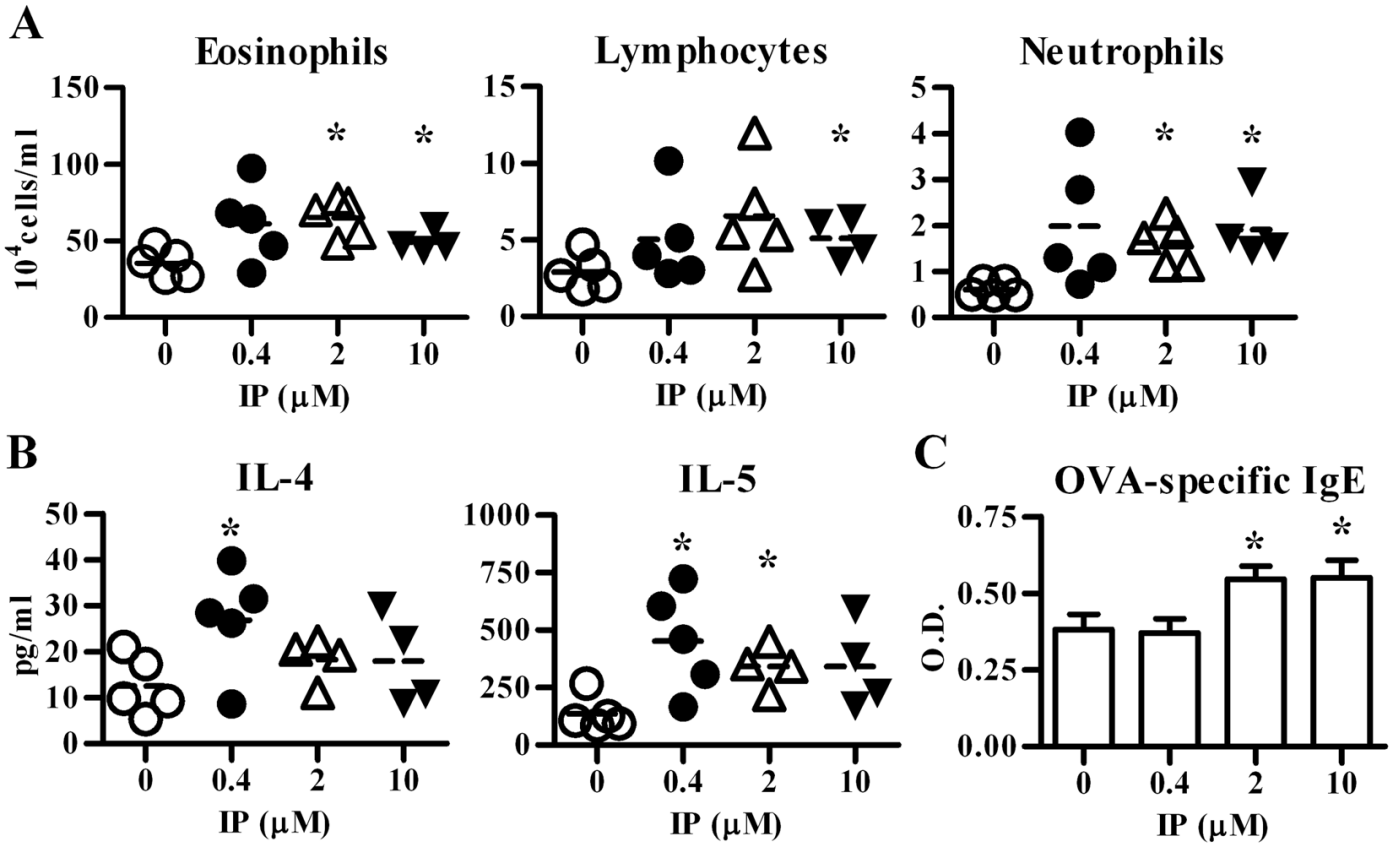
Indeno[1,2,3-cd]pyrene (IP) exposure enhances the severity of allergic lung inflammation in a mild asthmatic murine model. C57BL/6 mice were intranasally exposed to vehicle or various concentrations of IP as indicated and then all immunized and challenged with OVA. Cell subsets (**A**) and cytokine levels (**B**) in BALFs were both determined by flow cytometry. The line within the vertical points marks the mean for each group. (**C**) Serum levels of OVA-specific IgE Abs (mean ± SEM) were measured by ELISA. Data show one of three independent experiments. Eosinophil number, IL-5 and IgE levels showed group differences by one-way ANOVA. *P < 0.05 vs. vehicle-treated group (Mann-Whitney U test).

To determine whether IP was able to modulate airway allergic inflammation, a murine model was designed to mimic human exposure. The results showed that intranasal exposure of IP at concentrations ranging between 0.4 – 2 μM significantly enhanced the severity of pulmonary allergic inflammation, including increased infiltration of eosinophils, lymphocytes, and neutrophils (Fig. 2A), as well as enhanced IL-4 and IL-5 levels in bronchoalveolar lavage fluids (BALFs) (Fig. 2B). In addition, the levels of serum ovalbumin (OVA)-specific IgE were significantly enhanced in mice exposed to IP in a dose-dependent manner (Fig. 2C). These data suggest that IP may act as an adjuvant to modulate the allergic inflammation.

**Figure 3:**
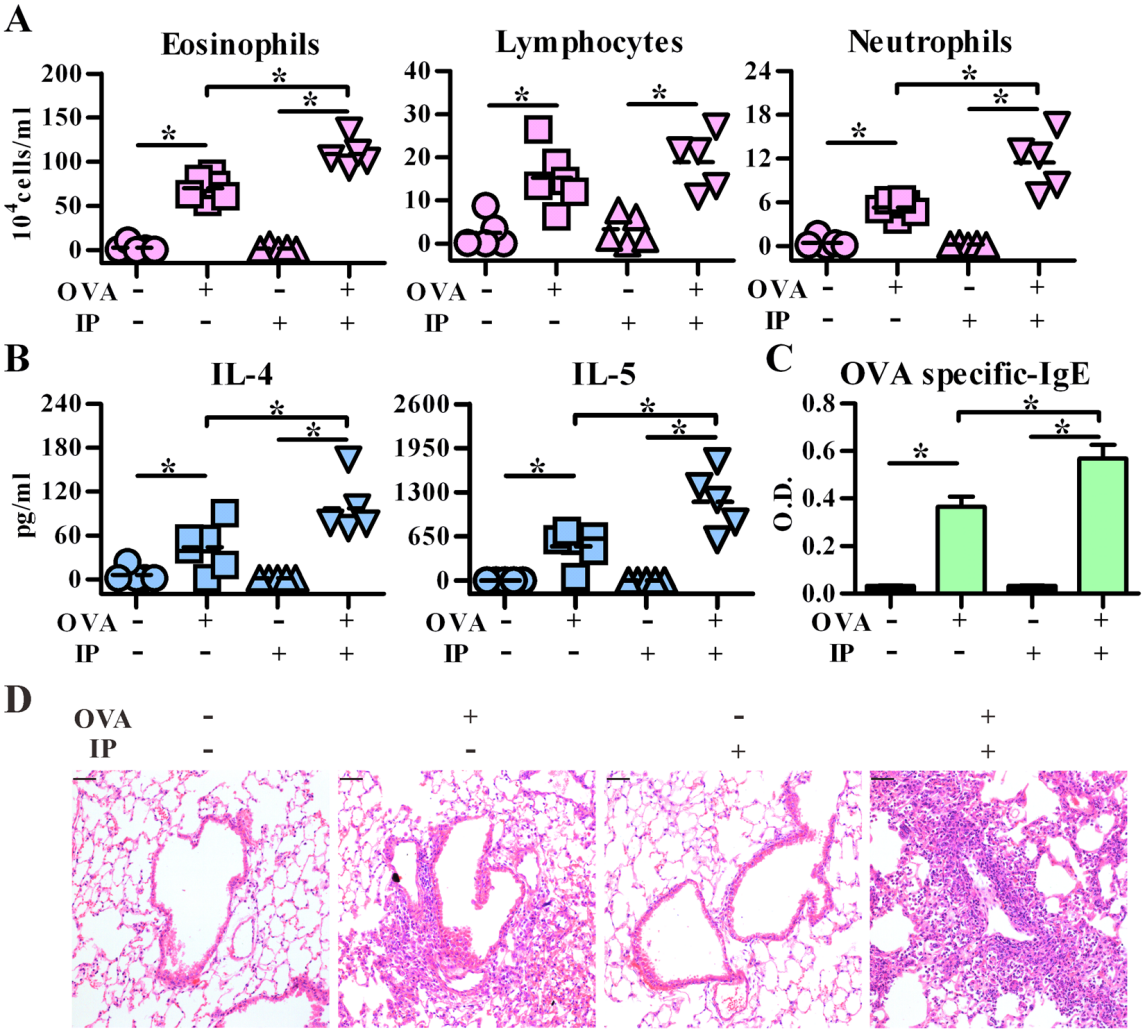
Indeno[1,2,3-cd]pyrene (IP) exposure aggravates the severity of allergic lung inflammation in the presence of allergen. C57BL/6 mice intranasally pre-exposed to vehicle or IP (2 μM) were then immunized either with PBS or OVA as indicated. All mice were then challenged with OVA aerosol for three consecutive days. Cell subsets (**A**) and cytokine levels (**B**) in BALFs were both determined by flow cytometry. The line within the vertical points marks the mean for each group. (**C**) Serum levels of OVA-specific IgE Abs (mean ± SEM) were measured by ELISA. (**D**) Representative lung sections stained with hematoxylin and eosin. The magnification: 200×. Data represent one of five independent experiments with consistent results. *P < 0.05 (Mann-Whitney U test). Scale bars represent 50 µm in (**D**).

In order to ensure the adjuvant role of IP in allergic responses, we tested the response induced by IP alone without antigen immunization. As shown in Fig. 3, IP exposure alone did not induce any allergic response, which was similar to vehicle control group (no OVA and no IP treatment). However, in the presence of OVA, IP exposure significantly enhanced OVA-induced allergic responses, including the levels of immune cell infiltration (Fig. 3A), Th2 cytokines (Fig. 3B) and OVA-specific IgE Abs (Fig. 3C). Further, immunocytochemistry analysis showed that while IP exposure alone did not induce apparent airway inflammation, prominent inflammatory lesions were noted in IP-treated, OVA-sensitized and challenged mice (Fig. 3D). These data suggest that airway IP exposure may act as an adjuvant to modulate immune responses towards Th2 responses, leading to the enhanced severity of allergic asthma.

### IP exposure modulated DC’s function in an AhR-dependent manner

**Figure 4:**
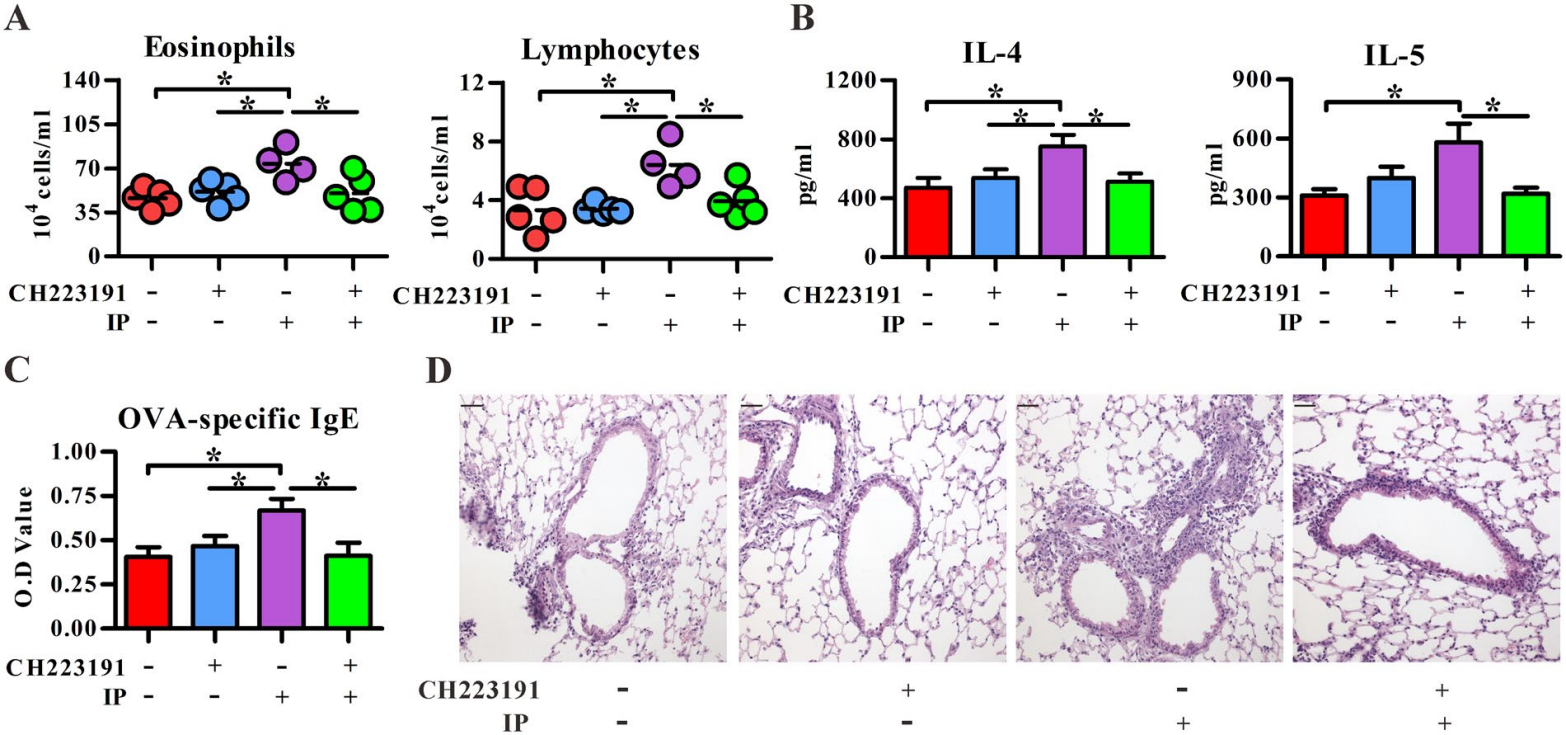
AhR antagonist inhibited the indeno[1,2,3-cd]pyrene (IP) effect on allergic lung inflammation. C57BL/6 mice intranasally exposed to methanol or IP (2 μM) in the presence ( + ) or absence (−) of AhR antagonist (CH223191, 10 μM) were immunized with OVA. All mice were challenged with OVA aerosol for three consecutive days. Cell subsets (**A**) or cytokine levels (mean ± SEM) (**B**) in BALFs were determined by flow cytometry or ELISA, respectively. (**C**) Serum levels of OVA specific IgE Abs (mean ± SEM) were measured by ELISA. (**D**) Representative lung sections stained with hematoxylin and eosin. The magnification: 200×. *P < 0.05 (Mann-Whitney U test). Data represent one of three independent experiments with consistent results in (**A**). Data are from 3 or 4 experiments in (**B**,**C**) (mean ± SEM), respectively. *n* = 7–9/group in (**B**); *n* = 12–13/group in (**C**). Scale bars represent 50 µm in (**D**).

**Figure 5:**
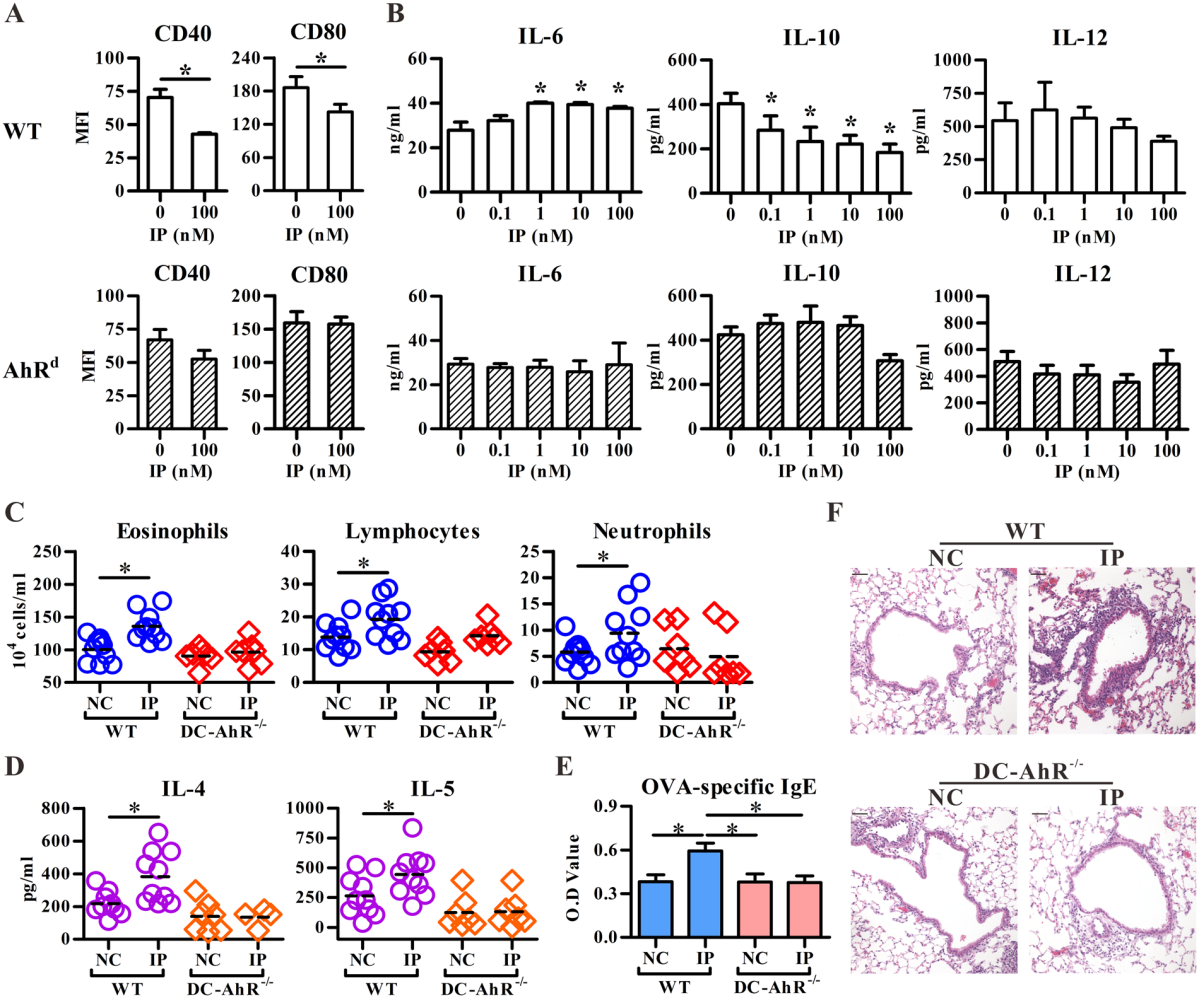
Indeno[1,2,3-cd]pyrene (IP) modulates DC function and allergic lung inflammation in an AhR-dependent manner. Bone marrow cells from C57BL/6 and AhR^d^ mice were treated with IP starting from day 1 to day 8, and LPS was added for another 24 hrs. The BM-DCs were harvested for phenotypic analysis (**A**) and the levels of cytokines in the culture supernatants were measured by ELISA (**B**). Data shows mean ± SD of three independent experiments. C57BL/6 (WT) or DC specific AhR-null mice (DC-AhR^−/−^) were intranasally pre-exposed to vehicle (NC) or IP (2 μM) and then immunized with OVA. All mice were challenged with OVA aerosol for three consecutive days. Cell subsets (**C**) and cytokine levels (**D**) in BALFs were both determined by flow cytometry. The line within the vertical points marks the mean for each group. (**E**) Serum levels (mean ± SEM) of OVA-specific IgE Abs were measured by ELISA. *n* = 7–10/group. (**F**) Representative lung sections stained with hematoxylin and eosin. The magnification: 200×. *P < 0.05 vs. control (Mann-Whitney U test). Scale bars represent 50 µm in (**F**).

IP has similar structure as BaP, which is a well-known ligand for AhR. Thus, we examined whether AhR was involved in IP-mediated effect on allergic lung inflammation. As shown in Fig. 4, administration of a commonly used AhR antagonist, CH223191, significantly inhibited the IP’s effect on OVA-induced influx of eosinophils and lymphocytes, Th2 cytokines, and OVA-specific IgE levels. Because DC plays an essential role in the pathogenesis of allergic asthma, we then asked whether IP modulated DC’s function through AhR by the use of C57BL/6 mice carrying a high-responder allele (AhR^b-1^) and a congenic strain carrying a low-responder allele of AhR (AhR^d^). The affinity of AhR^d^ for its ligand is 10–100 times lower than of AhR^b-1^ allele^25^. As shown in Fig. 5A and B, IP treatment at relatively low doses starting from 0.1 to 100 nM significantly inhibited LPS-induced expression of CD40, CD80 and IL-10 production, but enhanced LPS-stimulated IL-6 secretion in BM-DCs. However, IP exposure lost its effect on BM-DCs from AhR^d^ mice; also, IP exposure did not affect the allergic parameters in OVA-immunized DC-specific AhR-null (DC-AhR^−/−^) mice compared to those in wild type mice (Fig. 5C–F). Taken together, these data suggest that IP exposure may disturb DC’s function in an AhR-dependent manner, leading to the enhanced allergic lung inflammation.

**Figure 6:**
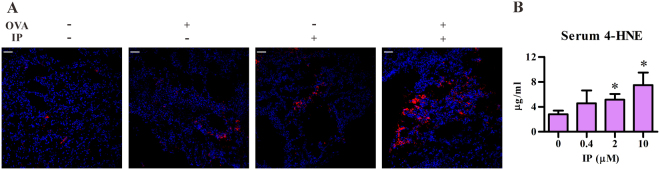
Detection of 4-HNE in lung tissues and in sera. (**A**) Frozen lung sections from treated mice as described in Fig. 3 were stained with DAPI (blue) and Cy3-conjugated 4-HNE Abs (red). The magnification: 200 × . (**B**) Serum levels of 4-HNE (mean ± SEM) in OVA immunized mice, as described in Fig. 2. Vehicle, *n* = 21; 0.4 μM, *n* = 8; 2 μM, *n* = 19; 10 μM, *n* = 7. Scale bars represent 50 µm in (**A**). *P < 0.05 vs. vehicle-treated group (Mann-Whitney U test).

It has been demonstrated that ultrafine particles associated with PAHs can induce oxidative stress and cytotoxicity in macrophages and epithelial cells^6^. Thus, we analyzed whether IP treatment promoted the production of 4-HNE, a sensitive oxidative stress marker, in the asthmatic murine model. As shown in Fig. 6, IP treatment in conjunction with OVA did enhance the 4-HNE generation in lung tissues as compared to those seen with IP or OVA treatment alone. Interesting, IP treatment alone without antigen challenge in mice also showed more 4-HNE-positive staining in lung tissues as compared to those in vehicle control group (no IP, no OVA treatment). Further, the level of 4-HNE in serum from OVA immunized mice was significantly increased upon IP exposure in a dose-dependent manner.

## Discussion

PAHs associated with air pollution have been considered as an important environmental risk for asthma^26^; however, the responsible component(s) of PAHs and the involved mechanism have not been well defined. In the present study, we identified IP as the main component of ambient PAHs in southern Taiwan, and showed its potential ability to enhance the severity of allergic lung inflammation through modulating DC’s function in an AhR-dependent manner. Further, biomonitoring analysis of a case-control study population showed that the urinary levels of OH-Py, a surrogate marker for PAH exposure, were significantly higher in patients with asthma than those seen in controls. This is the first study, to our knowledge, in providing evidence supporting a causal relationship between airborne IP exposure and lung inflammation using a mouse model of asthma.

The sources of IP were identified mainly from traffic^27^. However, as Kaohsiung City is an industrial city with steel plants, coal and coke combustion should also be considered as a source of the presence of IP in PM_2.5_^7^. Other atmospheric exposure of IP could include cooking related activities. For example, the concentration of IP in kitchen indoor air was found to be above the permissible Occupational Safety and Health Administration (OSHA) norms for indoor air^28^. Even though IP was found present in relatively higher amounts in many environmental matrices, the study of its toxicity has been scarce. While the toxic equivalent factor (TEF) with respect to BaP of IP has been assessed as 0.1^29^, the functional effect of IP on DCs appears to be more potent than that of BaP. BaP has been shown to inhibit DC maturation and IL-12 secretion in murine BM-DCs^30^ and in human monocyte-derived DCs^20^ at the concentrations of 1 and 10 μM, respectively. However, in our study, IP significantly suppressed IL-10 production and maturation in murine BM-DCs at the concentration of 0.1 nM and 100 nM, respectively (Fig. 5A,B). In addition, at least during the period between 2012 and 2013 in southern Taiwan, the amount of IP was around 4-fold higher than BaP in ambient air PAHs (Fig. 1C). It suggests, therefore, that IP, in addition to BaP, among PAHs associated with PM_2.5_, may contribute to the impact of air pollution on human health.

It has been noted that two prototypical AhR ligands, TCDD and FICZ, were shown to be able to modulate DCs with tolerogenic phenotypes and facilitate Foxp3^+^ regulatory T cell differentiation at relatively higher doses^22^. However, in our study, chronic exposure of IP at human exposure dosages in mice showed enhanced levels of pulmonary allergic inflammation. While it is currently unclear as to the reason why AhR’s ligands mediate these seemingly dichotomized immune responses, it may be due, in part, to a potential ligand-specific effect or, more than likely, different doses used in various studies. Consistent with our findings, Zhou *et al*. used relatively low-doses of AhR ligands, TCDD and FICZ, and showed enhanced mast cell response, including degranulation and cytokine generation^31^, while at higher doses following repetitive administration, FICZ (100 μg/kg/mouse) may have an inhibitory effect on mast cell’s response^32^, It is likely, therefore, that low doses of AhR’s ligand exposure may mediate immune activation, rather than tolerance. In fact, it is known that AhR ligand’s effect does not conform to the monotonic response curve. Nevertheless, further studies are needed to define the molecular basis of the AhR-ligand axis and its impact on immune system.

4-HNE is a reactive lipid mediator, which is generated from lipid peroxidation. Due to its electrophilic property, 4-HNE can form protein as well as lipid adducts and then modulate a number of signaling processes and impact cellular functions (reviewed in reference^33^). In the present study, 4-HNE was observed in the lung tissue of intranasally IP treated alone mice, suggesting exposure to PAHs may result in oxidative stress in the lungs. Although IP alone treated mice did not show obvious local allergic inflammation, pre-exposure to IP through airway significantly enhanced the severity of OVA-induced lung inflammation. These data suggest that exposure to environmental PAHs at human relevant exposure ways and doses is not expected to generate a dramatic “toxic” effect, but rather it may persistently modify the level of the host response. As a corollary, a recent study by Perzanowski *et al*. showed that prenatal exposure to cockroach allergen was associated with a greater risk of allergic sensitization in children and this risk was enhanced by exposure to PAHs^34^. These data support the link between air pollution and the severity of asthma in humans^35^.

Our study demonstrated that IP may act through AhR to alter DC function, and lead to enhanced allergic inflammation. As shown in Fig. 4, AhR antagonist CH223191 could reverse the IP effect on allergic lung inflammation, including inflammatory cell infiltration in BALFs and tissue, Th2 cytokine production and OVA-specific IgE levels. In addition to conventional DCs, several AhR-expressing cell types may also contribute to the development of IP-enhanced allergic inflammation in airway, such as epithelial cells^36^, fibroblast^37^, innate lymphoid cells^38^, mast cells^15,31^ as well as pDCs^39^. However, conventional DCs seem to play the major role in the IP’s effect because chronic airway exposure to IP in DC-AhR^−/−^ mice did not enhance the allergic lung inflammation in the OVA-induced asthma model. As IL-10 is a well-characterized immune suppressive cytokine, decreased expression of IL-10 in DCs may contribute to the IP’s effect on allergic inflammation. Moreover, two likely, but nor exclusive, mechanisms could be operated in IP’s potentiating effect. One is that airway DCs can be directly and chronically exposed to IP, which results in alteration of DC’s function as they carry the antigen and are capable of modulating the immune response via trafficking between epithelial cells and draining lymph nodes^40,41^. The other possible mechanism is that IP firstly acts on airway epithelium, which induces epithelial cells to release mediators and modulate local DC function. This evidence was provided by our cooperative study by Wang *et al*.^42^. The authors demonstrated that intranasal exposure to IP was sufficient to induce the generation of a lipid mediator, sphingosine-1-phosphate, from either mast cells, or epithelial cells or the combination of both without allergen sensitization in a mouse model, while sphingosine-1-phosphate is a potent bioactive lipid mediator that regulates lymphocyte trafficking, cell growth, apoptosis, inflammation and, perhaps, DCs^43^. In support of this notion, we also found that intranasal IP exposure alone without any allergen sensitization resulted in oxidative stress response in the lungs (Fig. 6A), but not inflammation (Fig. 3D), and as the consequence, significantly enhanced severity of OVA-induced airway inflammation was noted (Fig. 3). This suggests that PAHs may potentiate the development of asthma through the AhR-ligand axis in priming immune response. However, the detailed mechanism regarding how IP-AhR axis down-regulates IL-10 expression in DCs and its detailed mechanisms on the regulation of allergic asthma await further in-depth studies.

In the present study, asthmatic subjects in southern Taiwan showed elevated environmental PAH exposure. Chronic exposure to IP, a prominent ambient PAH, may affect DC functions through modulating AhR activity, leading to enhanced allergic inflammation in an established asthma murine model. Our study provides evidence supporting a direct link between ambient PAH exposure and the expression of allergic diseases.

## Methods

### Air sampling and PAH detection

One year air sampling campaign was carried out in four sampling sites in Kaohsiung City, Taiwan. The details of sampling and measurement of PAHs in PM_2.5_ are presented in reference^27^. Briefly, the 24-hr PM_2.5_ samples (twice a month, on 200 mm × 250 mm quartz fiber filter) were collected sequentially at each of the four sampling sites during the period of December 2012 to November 2013. The four sampling sites are SG, located in southern part of Kaohsiung with petrochemical, shipbuilding, firepower plant, iron, and steel industrial activities; FS, located in the northern part with petrochemical industrial activities nearby and densely populated; AG, located in the center of the city with intense traffic and commercial activities; while NSYSU is a coastal area site.

For PAH analysis, the quartz fiber filters were extracted with dichloromethane using an accelerated solvent extractor (Dionex ASE 300). The extracts were concentrated to 0.5 mL for GC-MS, a capillary gas chromatograph (Agilent 6890 N) and a mass spectrometer (Agilent 5973 N), analysis. Sixteen PAHs (USEPA priority) were determined, including naphthalene (Nap), acenaphthylene (Aceny), acenaphthene (Acen), fluorene (Flu), phenanthrene (Ph), anthracene (An), fluoranthene (Flt), pyrene (Py), benzo[a]anthracene (BaA), chrysene + triphenylene (Chry + TriPhe), benzo[b]fluoranthene (BbF), benzo[k]fluoranthene (BkF), BaP, IP, dibenzo[a,h]anthtracene (DBA), and benzo[g,h,i]perylene (BghiP).

### Patients

The study population included adult patients with current asthma (*n* = 39) at the outpatient departments of 8 medical centers following established protocols. We also enrolled normal control subjects (*n* = 43) selected from volunteers and from those who requested annual physical examinations without respiratory diseases. All subjects were non-smokers. All eligible subjects were enrolled in the study after signing the informed consent approved by the respective recruitment hospitals. Patients with age ≥18 years were enrolled and met the following inclusion criteria: (1) at least 18 years of age, (2) physician-diagnosed asthma. Physician’s diagnosis of asthma was made according to the 2008 Global Initiative for Asthma (GINA) guidelines. In this study, we further stratified asthma into four groups: asthma that required GINA Step 1 or 2 to maintain good control was considered as mild asthma, GINA Step 3 as moderate, GINA Step 4 as severe (Table 1). Patients with severe asthma (step 4) were receiving more than two combination controller therapies (ICS, LABA, leukotriene modifier and sustained-release theophylline). Patients were also evaluated for their control status by using asthma control test (ACT), a validated patient-completed questionnaire consisting of five parameters aimed at assessing asthma symptoms (daytime and nocturnal), use of rescue medications, and the effect of asthma on daily functioning. The scores range from 5 (poor control of asthma) to 25 (complete control of asthma). The scores equal or less than 19 was considered to be “not well controlled”. Pulmonary function was measured with a Jaeger Master screen Pulmonary System spirometer (Hoechberg, Germany). At baseline, FEV1 and FVC were expressed as percentages of the predicted values, whereas FEV1/FVC was reported only as a ratio. The smoking status of all patients was also assessed and divided into three groups: current smokers, ex-smokers who had stopped smoking for a minimum of 1 month before the initial visit, and lifetime non-smokers. Patients have one of self-reported allergic triggers (pollen, mold, dust, animals, beans, seafood, milk and eggs), allergic rhinitis and/or atopic dermatitis were defined as having positive history of allergy. Urine samples were collected and stored frozen in glass container until analysis. In this dataset, demographical comparison revealed significant differences in second-hand smoke at home and lung function parameters (FEV1 and FEV1/FVC ratio) between controls and patients (Table 1). The study protocol was approved by the Institutional Review Boards of Kaohsiung Medical University Hospital (KMU-IRB-990392). The study was performed in accordance with the ethical standards laid down in the Declaration of Helsinki.

**Table 1:** Demographic characteristics of patients with asthma and normal controls.

	Asthma (*n* = 39)	Normal (*n* = 43)	P value
Gender, *n* (%)			0.658
Male	20 (51.3)	21 (48.8)	
Female	19 (48.7)	22 (51.23)	
Age (mean ± SD)	55 ± 13	57 ± 11	0.454
BMI (mean ± SD)	25 ± 4	24 ± 4	0.602
Smoking habits, *n* (%)			0.241
Never-smokers	30 (76.9)	38 (88.3)	
Ex-smokers	9 (23.1)	5 (11.7)	
Second hand smoke at home, *n* (%)			0.037*
Absent	18 (46.1)	10 (23.3)	
Present	21 (53.9)	33 (76.7)	
Second hand smoke at work, *n* (%)			0.497
Absent	26 (66.7)	25 (58.1)	
Present	13 (33.3)	18 (41.9)	
FEV1% predicted (mean ± SD)	71.6 ± 22.5	87.2 ± 11.6	0.0003*
FEV1/FVC (mean ± SD)	68.4 ± 11.5	86.0 ± 9.1	1.4×10^−10^*
Severity, *n* (%)			
Mild (GINA 1,2)	20 (51.3)		
Moderate (GINA 3)	11 (28.2)		
Severe (GINA 4)	8 (20.5)		
Allergy history, *n* (%)			0.183
Absent	18 (46.2)	27 (62.8)	
Present	21 (53.8)	16 (37.2)	
ACT score (mean ± SD)	20.6 ± 4.0		
Total IgE level (KU/L, mean ± SD)	247.5 ± 426.3		
Eosinophil (per mm^3^, mean ± SD)	249.8 ± 182.1		
Urine creatinine (mg/dL, mean ± SD)	96.5 ± 69.2	104.6 ± 45.1	0.528

^*^Statistically significant (*P* < 0.05).

ACT = asthma control test; BMI = body mass index; FEV1 = forced expiratory volume; FVC = forced vital capacity.

### Urinary PAH metabolite detection

Urinary OH-Py was measured with previously published assay^44^ and used as a surrogate marker for the exposure of PAHs, as the standard IP metabolite was not commercially available at the time of analysis. First, the defrosted urine (10 mL) in acetate buffer (0.1 M acetic acid and 0.1 M sodium acetate) was digested with α-glucuronidase/aryl-sulphatase, and then OH-Py in the urine samples was extracted, enriched and purified by cartridges packed with C-18 reversed-phase liquid chromatographic material (Waters SEP-PAK VAC C-18, Waters, Milford, MA, USA) at a loading rate of less than 3 mL/min. The retained solutes in cartridges were eluted with 6 mL of isopropyl alcohol and dried. The processed sample was dissolved in 2 mL of isopropyl alcohol for analysis, using a high-performance liquid chromatography (Waters 2695) with fluorescence detector (Waters 474) and a 150 mm × 4 mm LiChrosorb RP-18 (5 mm) column (Supelo). The mobile phase was 65% methanol (methanol/water 1⁄4 65:35). The excitation wavelength and emission wavelength of fluorescence were 281 and 388 nm, respectively. This method had a limit of detection of 0.028 ng/mL, with an average recovery rate of 96.18%, and a coefficient of variance smaller than 10% for repeated measurements. The urinary levels of OH-Py were normalized by the levels of urinary CRN and expressed as μg/g CRN.

### Chemicals

IP was purchased from Sigma–Aldrich (St. Louis, Mo., USA) and stocked in methanol.

### Mice

The protocol used in all animal experiments was approved by Institutional Animal Care and Use Committee of the Kaohsiung Medical University (Permit Number: 101048), and was in accordance with the guidelines and regulations of the institution. Female C57BL/6 and AhR^d^ congenic mice, aged 6–8 weeks were obtained from National Laboratory Animal Center, Taiwan. DC-AhR^−/−^ mice were generated by crossing CD11c^cre^ and AhR^fl/fl^ mice, which were both from National Health Research Institutes. All mice maintained in a pathogen-free facility.

### Establishment and assessment of allergic lung inflammation murine model

Different concentrations of IP (0.4, 2 or 10 μM) or 0.2% methanol (vehicle) were intranasally delivered into naïve female mice every other day between day 1 and day 20. The mice received OVA (20 μg/mouse) or PBS plus Al(OH)_3_ intraperitoneally on day 7 and then daily received 3% OVA aerosol exposure on days 18–20. On day 21, BALFs were collected and the cells in BALFs were stained with PE-Cy7-anti-CD11c (N418; eBioscience), FITC-anti-I-A^d^/I-E^d^ (M5/114.15.2; eBioscience), PE-anti-CCR3 (83101; R&D Systems), APC-anti-CD3 (145–2C11; BD Biosciences, San Diego, California, USA) and anti-B220 (RA3–6B2; eBioscience) antibodies (Abs)^45^. The cellular composition of BALF cells was determined by flow cytometry (LSR II; BD Biosciences).

### Cytokine determination

Cytokine levels in supernatants from BM-DC cultures or in BALFs from treated mice were assessed by ELISA (eBioscience, Ireland, UK) or cytokine beads array (LEGENDplex; BioLegend) according to manufacturers’ instructions, respectively.

### OVA-specific IgE detection

The levels of OVA-specific IgE in serum samples were determined by a standard ELISA method. Briefly, five-fold diluted serum was loaded in OVA-coated 96 well plate, and followed by biotin rat anti-mouse IgE (R35-72, BD biosciences), avidin-horseradish peroxidase, and tetramethylbenzidine substrate. The absorbance (O.D value) was measured by VERSAmax ELISA reader (Molecular Devices, Sunnyvale, California., USA) at 450 nm and corrected by 540 nm.

### Lung pathology

The whole lung was fixed in 3.7% formaldehyde and embedded in paraffin. The tissue sections (3 μM) were stained by hematoxylin and eosin, according to the manufacture’s protocol (NovoLink^TM.^ Polymer Detection System, Leica, UK). Frozen samples embedded in OCT (5 μM) were stained with DAPI or rabbit anti-4-hydroxynonenal (4-HNE) Ab (Abcam, UK) followed by goat anti-rabbit IgG Alexa Fluor 568 (Invitrogen) according to manufacturers’ instructions. The level of 4-HNE in serum was analyzed using mouse specific 4-HNE ELISA kit (OxiSelect HNE Adduct Competitive ELISA kit, Cell Biolabs, CA, USA), according to manufacturers’ instructions.

### BM-DCs generation and treatment

BM-DCs were prepared as described previously^46^. Briefly, bone marrow cells were cultured with recombinant murine GM-CSF (rmGM-CSF) (500 U/ml, Pepro Tech Inc., Rocky Hill, NJ) and treated with various concentrations of IP or 0.2% methanol (as a vehicle control) at the beginning of the day 1. The medium containing rmGM-CSF and IP or methanol was refreshed on day 3 and day 6. On day 8, BM-DCs were stimulated with or without LPS (1 μg/ml, Escherichia coli O127:B8; Sigma-Aldrich) for another 24 hrs. The phenotype of BM-DCs were analyzed by flow cytometry (LSR II; BD Biosciences) for the expression of CD11c (G418), CD40 (1C10), CD80 (16-10A1), CD86 (GL1) and MHC class II (M5/114.15.2).

### Statistical Analysis

The independent t-tests were used to examine the differences of age, body mass index, and urine CRN. Chi-square tests were used to compare the difference of gender, smoking habits, and second-hand smoke status. One-way ANOVA was used to compare the difference among groups. Nonparametric Mann-Whitney U tests were conducted to examine the differences of human urinary OH-Py and the results from control and treated cells or mice. P values < 0.05 were considered significant. All statistical tests were performed by SPSS for Windows, version 22.0. (SPSS Inc., Chicago, Ill., USA).
